# Complete fatty acid analysis data of flaxseed oil using GC-FID method

**DOI:** 10.1016/j.dib.2019.103845

**Published:** 2019-03-19

**Authors:** Mohammed Danish, Maniruddin Nizami

**Affiliations:** a Green Chemistry & Sustainable Engineering Technology Research Cluster, Bioengineering Section, Universiti Kuala Lumpur Malaysian Institute of Chemical and Bioengineering Technology, Lot 1988, KPBV, Taboh Naning, 78000 Alor Gajah, Melaka, Malaysia; b Australian Laboratory Services Arabia Co. Ltd., PO Box 9692, Dammam 31423, Saudi Arabia

**Keywords:** Flaxseed oil, Fatty acids, Gas chromatography, Trans-fats, Fatty acid methyl ester (FAME)

## Abstract

The data presented in this article were generated through the gas chromatography (GC) with a flame ionization detector (FID). The flaxseed oil was converted into fatty acid methyl ester (FAME) then used in the GC with FID and observe the retention time of different fatty acid present in the flaxseed oil. The observed retention time was compared with the standard fatty acid to confirm the specific fatty acid presence in the flaxseed oil. The part of the data is used in the article “Optimization of the process variable for biodiesel production by transesterification of flaxseed oil and produced biodiesel characterizations” Renewable Energy journal (Ahmad et al., 2019).

**Table:** Specifications table

Subject area	*Analytical chemistry*
More specific subject area	*Food analysis*
Type of data	*Table, image, text file, graph, figure*
How data was acquired	*Gas chromatography with a flame ionization detector, model of the instrument: Agilent GC 7890B with Flame Ionization Detector.*
Data format	*Raw, filtered, analyzed.*
Experimental factors	*Flaxseed oil pretreated with methanolic NaOH, BF*_*3*_*, and filter through sodium sulfate.*
Experimental features	*Conversion of fatty acids into Fatty acid methyl ester (FAME) was an essential feature of the GC-FID analysis.*
Data source location	*Dammam, Saudi Arabia.*
Data accessibility	*All data related to the fatty acid analysis of flaxseed oil is included in this article.*
Related research article	“Optimization of process variable for biodiesel production by transesterification of flaxseed oil and produced biodiesel characterizations” Renewable Energy Journal [1].

**Table:** 

**Value of the data** • The data give complete information about the fatty acids present in the flaxseed oil. • Standard of each fatty acid data can be used for the reference for the other oil analysis. • The percentage information of the saturated fats (SAFA), Trans-fats (TFA), Monounsaturated fats (MUFA), polyunsaturated fats (PUFA), and total unsaturated fats (TUFA) in flaxseed oil will help researchers in food and nutrition. • Fatty acid profiling of the oil will help in converting flaxseed oil into biodiesel.

## 1 Data

**Fig. 1:**
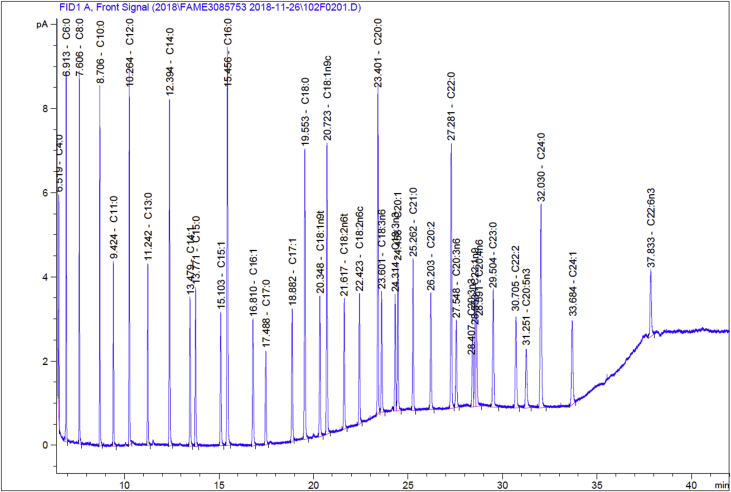
GC-FID chromatogram of supelco 37 component FAME mix, Cat: CRM47885, Lot: XA19807V.

**Fig. 2:**
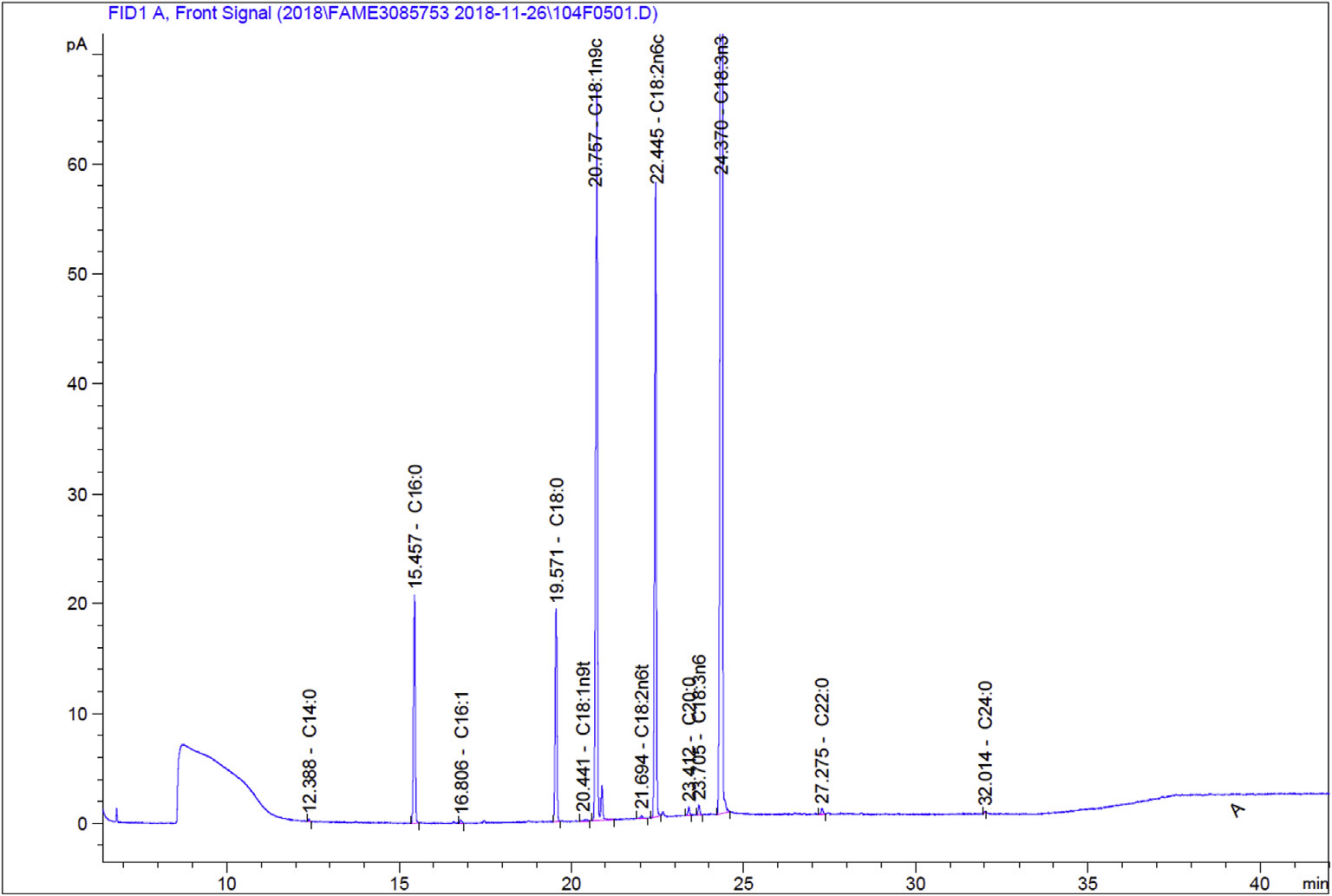
GC-FID Chromatogram of flaxseed oil with peak label (Retention Time & Name of Fatty Acid).

The data collected for fatty acid are given in the below tables. Table 1 represents the standard Supelco 37 FAME data for comparison purpose. Table 2 shows the GC-FID data for flaxseed oil converted FAME. The chromatogram of standard Supelco 37 is shown Fig. 1 and flaxseed oil converted FAME chromatogram is shown in Fig. 2.

**Table 1:** Supelco 37 component FAME Mix FAME analysis data used for calibration for quantitation & identification of the unknown peaks in the oil samples.

SN	Fatty acids in CRM	Fatty acid groups	RT	Short name	% Area
1	C4:0–Butyric acid	SAFA	6.519	C4:0	1.69614
2	C6:0–Caproic acid	SAFA	6.913	C6:0	3.13618
3	C8:0–Caprylic acid	SAFA	7.606	C8:0	3.22269
4	C10:0–Capric acid	SAFA	8.706	C10:0	3.3805
5	C11:0–Undecanoic acid	SAFA	9.424	C11:0	1.84709
6	C12:0–Lauric acid	SAFA	10.264	C12:0	3.94123
7	C13:0–Triundecanoic acid	SAFA	11.242	C13:0	2.06048
8	C14:0–Myristic acid	SAFA	12.394	C14:0	4.32965
9	C14:1–Myristoleic acid	MUFA	13.479	C14:1	2.13117
10	C15:0–Pentadecanoic acid	SAFA	13.771	C15:0	2.23474
11	C15:1–*cis*-10-Pentadecenoic acid	MUFA	15.103	C15:1	2.15963
12	C16:0–Palmitic acid	SAFA	15.456	C16:0	6.3978
13	C16:1–Palmitoleic acid	MUFA	16.810	C16:1	2.24513
14	C17:0–Heptadecanoic acid	SAFA	17.488	C17:0	1.56535
15	C17:1–*cis*-Heptadecenoic acid	MUFA	18.882	C17:1	2.21837
16	C18:0–Stearic acid	SAFA	19.553	C18:0	4.69103
17	C18:1–*trans*-9-Elaidic acid	TFA	20.348	C18:1n9t	2.28174
18	C18:1 (n-9)–Oleic acid	MUFA/ω9FA	20.723	C18:1n9c	4.5848
19	C18:2–*trans*-Linolelaidic acid	TFA	21.617	C18:2n6t	2.10519
20	C18:2 (n-6)–Linoleic acid	PUFA	22.423	C18:2n6c	2.09427
21	C20:0–Arachidic acid	SAFA	23.401	C20:0	4.68651
22	C18:3 (n-6)–g-Linolenic acid	PUFA/ω6FA	23.601	C18:3n6	1.91131
23	C18:3 (n-3)–a-Linolenic acid (ALA)	PUFA/ω3FA	24.314	C18:3n3	1.84683
24	C20:1 (n-9)–*cis*-11-Eicosenic acid	MUFA	24.458	C20:1	2.3618
25	C21:0–Heneicosanoic acid	SAFA	25.262	C21:0	2.36044
26	C20:2–*cis*-11,14-Eicosadienoic acid	PUFA	26.203	C20:2	2.07894
27	C22:0–Behenic acid	SAFA	27.281	C22:0	4.67455
28	C22:3n6–*cis*-8,11,14-Eicostrienoic acid	PUFA/ω6FA	27.548	C20:3n6	1.69522
29	C20:3n3–*cis*-11,14,17-Eicosatrienoic acid	PUFA/ω3FA	28.407	C20:3n3	1.38465
30	C22:1 (n-9)–Erucic acid	MUFA/ω9FA	28.553	C22:1n9	2.32792
31	C20:4 (n-6)–Arachidonic acid	PUFA/ω6FA	28.591	C20:4n6	1.74384
32	C23:0–Tricosanoic acid	SAFA	29.504	C23:0	2.46567
33	C22:2–*cis*-13,16-Docasadienoic acid	PUFA	30.705	C22:2	2.114
34	C20:5 (n-3)–*cis*-5,8,11,14,17-Eicosapentaenoic acid (EPA)	PUFA/ω3FA	31.251	C20:5n3	1.53761
35	C24:0–Lignoceric acid	SAFA	32.030	C24:0	4.75565
36	C24:1–Nervonic acid	MUFA	33.684	C24:1	2.14213
37	C22:6 (n-3)–*cis*-4,7,10,13,16,19-Docosahexaenoic acid (DHA)	PUFA	37.833	C22:6n3	1.58971

**Table 2:** Fatty acids with their relative percentage in the total fat of the flaxseed oil.

SN	Fatty acids in flaxseed oil	Fatty acid groups	RT	Peak area (FAME)	% Fat (of total fat)
1	C14:0–Myristic acid	SAFA	12.388	0.569951	0.046
2	C16:0–Palmitic acid	SAFA	15.457	70.39929	5.687
3	C16:1–Palmitoleic acid	MUFA	16.806	1.19302	0.096
4	C18:0–Stearic acid	SAFA	19.571	68.69044	5.578
5	C18:1–*trans*-9-Elaidic acid	TFA	20.441	1.06464	0.086
6	C18:1 (n-9)–Oleic acid	MUFA/ω9FA	20.757	253.6431	20.591
7	C18:2–*trans*-Linolelaidic acid	TFA	21.649	1.10871	0.09
8	C18:2 (n-6)–Linoleic acid	PUFA/ω6FA	22.445	194.69879	15.801
9	C20:0–Arachidic acid	SAFA	23.412	2.50581	0.204
10	C18:3 (n-6)–g-Linolenic acid	PUFA	23.705	2.87935	0.234
11	C18:3 (n-3)–a-Linolenic acid (ALA)	PUFA/ω3FA	24.37	633.32971	51.376
12	C22:0–Behenic acid	SAFA	27.275	2.17424	0.178
13	C24:0–Lignoceric acid	SAFA	32.014	0.394852	0.034
14	Sum of Omega-3 (n-3)	ω3FA	–	–	51.376
15	Sum of Omega-6 (n-6)	ω6FA	–	–	15.801
16	Sum of Omega-9 (n-9)	ω9FA	–	–	20.591
17	Saturated fats (SAFA)	SAFA	–	–	11.727
18	Trans-fats (TFA)	TFA	–	–	0.176
19	Monounsaturated fats (MUFA)	MUFA	–	–	20.687
20	Polyunsaturated fats (PUFA)	PUFA	–	–	67.41
21	Total Unsaturated fats (TUFA)	TUFA	–	–	88.097

ω3FA = Omega-3 Fatty Acids, ω6FA = Omega-6 Fatty Acids, ω9FA = Omega-9 Fatty Acids, SAFA = Saturated Fatty Acids, TFA = Trans Fatty Acids, MUFA = Monounsaturated Fatty Acids, PUFA = Polyunsaturated Fatty Acids, TUFA = Total Unsaturated Fatty Acids.

## 2 Experimental design, materials, and methods

### 2.1 FAME preparation from flaxseed oil

**Scheme 1:**
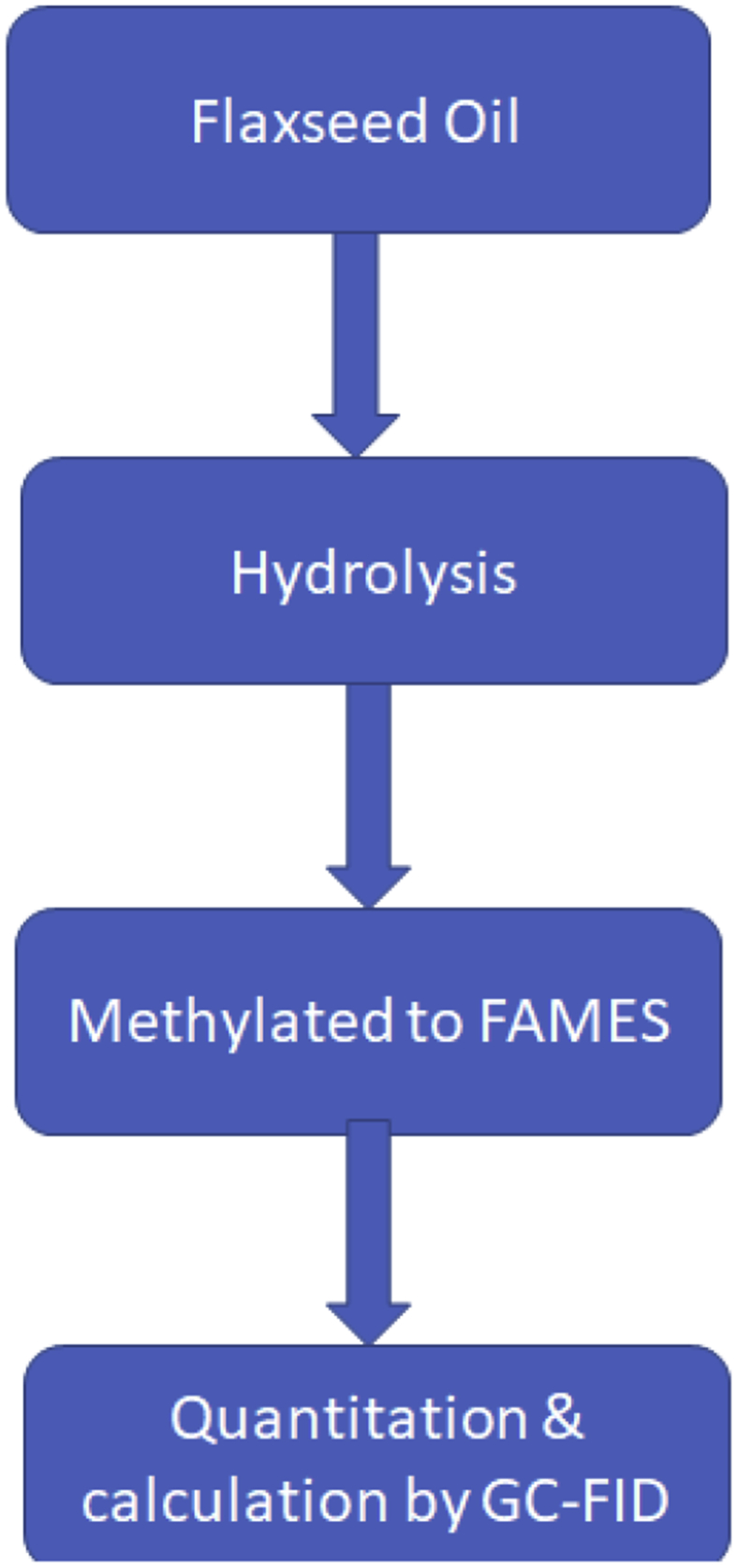
Flow chart of the flaxseed oil fatty acid analysis.

For conversion of flaxseed oil into FAME the following methods was adopted: around 0.1 g flaxseed oil was taken into 40 mL glass vial then mix with 5 mL of 0.50 N methanolic NaOH (Methanol: VWR Chemicals, 20864.320, Batch 14C030509. NaOH: PanReac, 141687.1211, Lot # 0001070723), the mixture was heated for 3 mins at 60 °C. The mixture was allowed to cool at room temperature, then 6 mL of 14% BF_3_ solution (Aldrich, B1250-500mL, Lot # BCBW8950) was added [2] to the mixture and again heated for 3 mins at 60 °C. The mixture was again cool at room temperature then added 10 mL isooctane (Carlo Erba, 412460 2.5 L) and shake it well, then keep it to settling down. After settling the mixture, the upper layer of the mixture was transferred to the tube containing sodium sulfate (Ajax Finechem, 503-500G, B/No. 1608224929) to remove the moisture. The extract was analyzed with GC-FID (Agilent 7890B), the flow chart of the flaxseed oil analysis method condition shown in Scheme 1 The quantity and identification of fatty acid in the flaxseed oil was done through the comparison of standard supelco 37 FAME component [3].

### 2.2 GC acquisition method

Following are the detail of the gas chromatography instrumentation and methods [4].

GC oven program: Initial Temperature: 120 °C, Hold Time: 1 min. Rate 1: 10 °C/min to 175 °C, Hold Time: 10 min. Rate 2: 5 °C/min to 210 °C, Hold Time: 10 min. Rate 3: 5 °C/min to 230 °C, Hold Time: 9.5 min.

Equilibration Time: 0.5 min. Max Temperature: 260 °C.

Automatic Liquid Sampler Injector: Syringe Size: 10 μL, Injection Volume: 1 μL, Injection Dispense Speed: 6000 μL/min, Viscosity Delay: 0 sec.

Sample inlet parameters: Split/Splitless Inlet. Mode: Split, Heater: 250 °C, Pressure: 20.863 psi, Total Flow: 54 mL/min, Septum Purge Flow: 3 mL/min, Split Ratio: 50:1, Split Flow: 50 mL/min.

Column Parameters: Initial Flow: 1 mL/min, Post Run: 1.4 mL/min.

Column Specifications: Agilent 112-88A7, HP-88, 0 °C - 250 °C (260 °C): 100 m × 250 μm x 0.2 μm.

Detector Parameters: Flame Ionization Detector (FID), Heater: 260 °C, H_2_ Flow: 40 mL/min, Air Flow: 450 mL/min, Makeup Flow: Off.
