# Supplementary material for: Complete fatty acid analysis data of flaxseed oil using GC-FID method
Source: Data Brief. 2019 Mar 19;23:103845. doi: 10.1016/j.dib.2019.103845 (PMC6661239; doi:10.1016/j.dib.2019.103845)
Supplement: Multimedia Component 2 [file mmc2.pdf]

Data File D:\Chem32\1\Data\2018\FAME3085753 2018-11-26\102F0201.D
Sample Name: Supelco 37 Component FAME STD; CRM47885, Lot: XA19807V

===

Acq. Operator : SYSTEM Seq. Line : 2
Acq. Instrument : Agilent 7890B GC_Food Laborat Location : 102 (F)
Injection Date : 11/26/2018 5:40:12 PM Inj : 1
Inj Volume : 1 µl
Acq. Method : D:\Chem32\1\Data\2018\FAME3085753 2018-11-26\FAMES_Agilen3.M
Last changed : 11/26/2018 4:48:48 PM by SYSTEM
Analysis Method : D:\CHEM32\1\DATA\2018\FAME3085753 2018-11-26\FAMES_Agilent2_Process.M
Last changed : 11/27/2018 8:00:03 AM by SYSTEM
Sample Info : Supelco 37 Component FAME STD; CRM47885, Lot: XA19807V

Additional Info : Peak(s) manually integrated

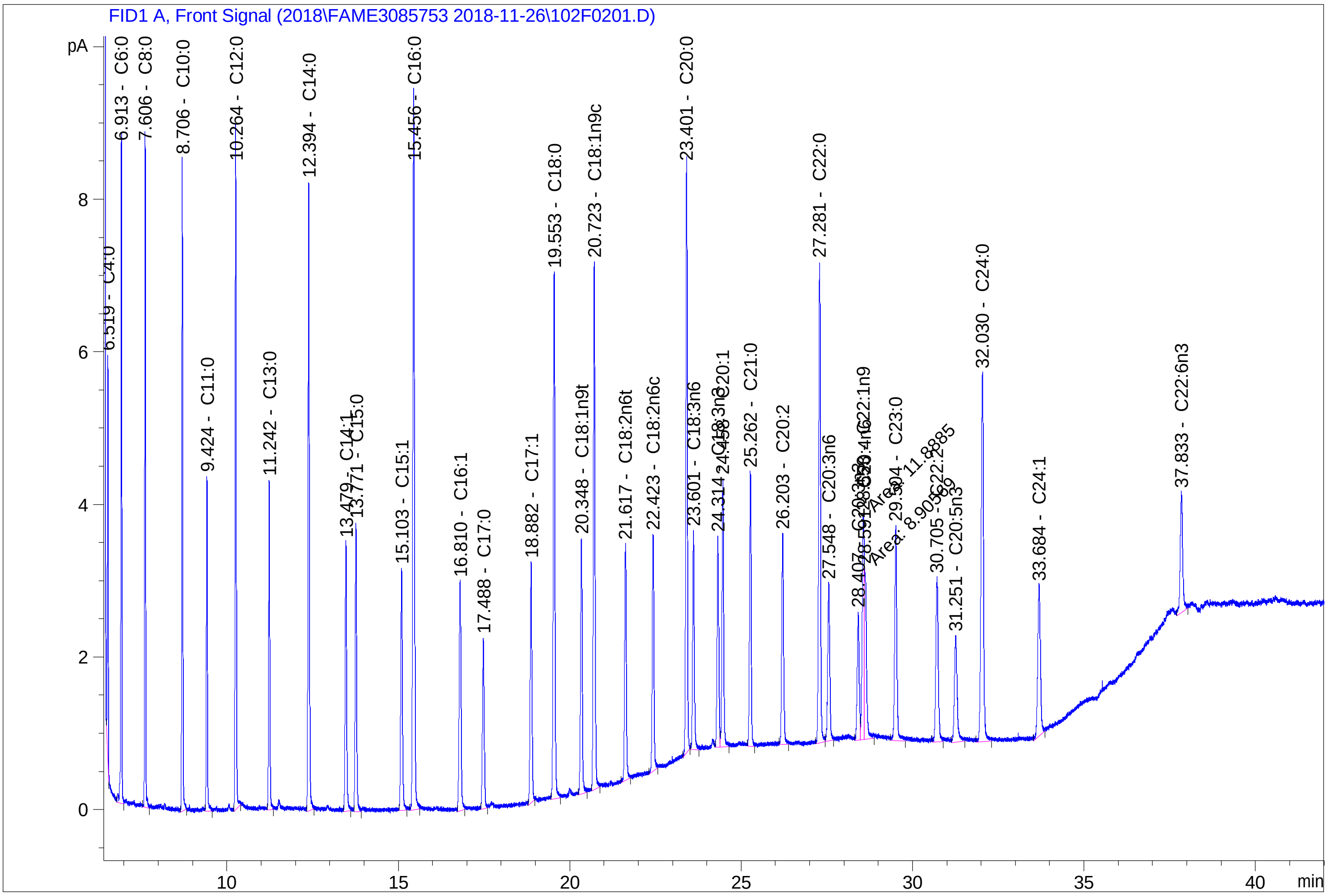

===

Area Percent Report

===

Sorted By : Signal
Calib. Data Modified : 11/26/2018 7:23:18 AM
Multiplier : 1.0000
Dilution : 1.0000
Do not use Multiplier & Dilution Factor with ISTDs

Signal 1: FID1 A, Front Signal

Peak #	RetTime [min]	Type	Width [min]	Area [pA*s]	Area %	Name
1	6.519	BB	0.0258	8.66207	1.69614	C4:0
2	6.913	BB	0.0277	16.01628	3.13618	C6:0
3	7.606	BB	0.0285	16.45803	3.22269	C8:0
4	8.706	BB	0.0312	17.26401	3.38050	C10:0
5	9.424	BB	0.0331	9.43296	1.84709	C11:0
6	10.264	BB	0.0345	20.12758	3.94123	C12:0
7	11.242	BB	0.0377	10.52275	2.06048	C13:0
8	12.394	BB	0.0413	22.11125	4.32965	C14:0
9	13.479	BB	0.0473	10.88374	2.13117	C14:1
10	13.771	BB	0.0469	11.41266	2.23474	C15:0
11	15.103	BB	0.0531	11.02910	2.15963	C15:1
12	15.456	BB	0.0535	32.67314	6.39780	C16:0
13	16.810	BB	0.0590	11.46571	2.24513	C16:1
14	17.488	BB	0.0563	7.99416	1.56535	C17:0
15	18.882	BB	0.0565	11.32908	2.21837	C17:1
16	19.553	BB	0.0542	23.95677	4.69103	C18:0
17	20.348	BB	0.0527	11.65271	2.28174	C18:1n9t
18	20.723	BB	0.0507	23.41428	4.58480	C18:1n9c
19	21.617	BB	0.0525	10.75107	2.10519	C18:2n6t
20	22.423	BB	0.0523	10.69532	2.09427	C18:2n6c
21	23.401	BB	0.0470	23.93369	4.68651	C20:0
22	23.601	BB	0.0526	9.76091	1.91131	C18:3n6
23	24.314	BV	0.0512	9.43161	1.84683	C18:3n3
24	24.458	VB	0.0530	12.06156	2.36180	C20:1
25	25.262	BB	0.0520	12.05463	2.36044	C21:0
26	26.203	BB	0.0569	10.61698	2.07894	C20:2
27	27.281	BV	0.0571	23.87261	4.67455	C22:0
28	27.548	VB	0.0619	8.65737	1.69522	C20:3n6
29	28.407	BV	0.0653	7.07132	1.38465	C20:3n3
30	28.553	MF	0.0674	11.88853	2.32792	C22:1n9
31	28.591	FM	0.0664	8.90569	1.74384	C20:4n6
32	29.504	BB	0.0683	12.59200	2.46567	C23:0
33	30.705	BB	0.0763	10.79607	2.11400	C22:2
34	31.251	BB	0.0861	7.85246	1.53761	C20:5n3
35	32.030	BB	0.0770	24.28679	4.75565	C24:0
36	33.684	BB	0.0862	10.93971	2.14213	C24:1
37	37.833	BB	0.0756	8.11854	1.58971	C22:6n3

Totals : 510.69312 100.0000

Uncalibrated Peaks: n.a.

1 Warnings or Errors :

Warning : Calibration warnings (see calibration table listing)

===

Compound-related custom fields:

*** End of Report ***
