# Supplementary material for: Complete fatty acid analysis data of flaxseed oil using GC-FID method
Source: Data Brief. 2019 Mar 19;23:103845. doi: 10.1016/j.dib.2019.103845 (PMC6661239; doi:10.1016/j.dib.2019.103845)
Supplement: Multimedia Component 4 [file mmc4.pdf]

===

Acq. Operator : SYSTEM Seq. Line : 1
Acq. Instrument : Agilent 7890B GC_Food Laborat Location : 101 (F)
Injection Date : 11/26/2018 4:50:10 PM Inj : 1
Inj Volume : 1 µl
Acq. Method : D:\Chem32\1\Data\2018\FAME3085753 2018-11-26\FAMES_Agilen3.M
Last changed : 11/26/2018 4:48:48 PM by SYSTEM
Analysis Method : D:\CHEM32\1\DATA\2018\FAME3085753 2018-11-26\FAMES_Agilent2_Process.M
Last changed : 11/27/2018 8:02:43 AM by SYSTEM
Sample Info : Metho Blank

Additional Info : Peak(s) manually integrated

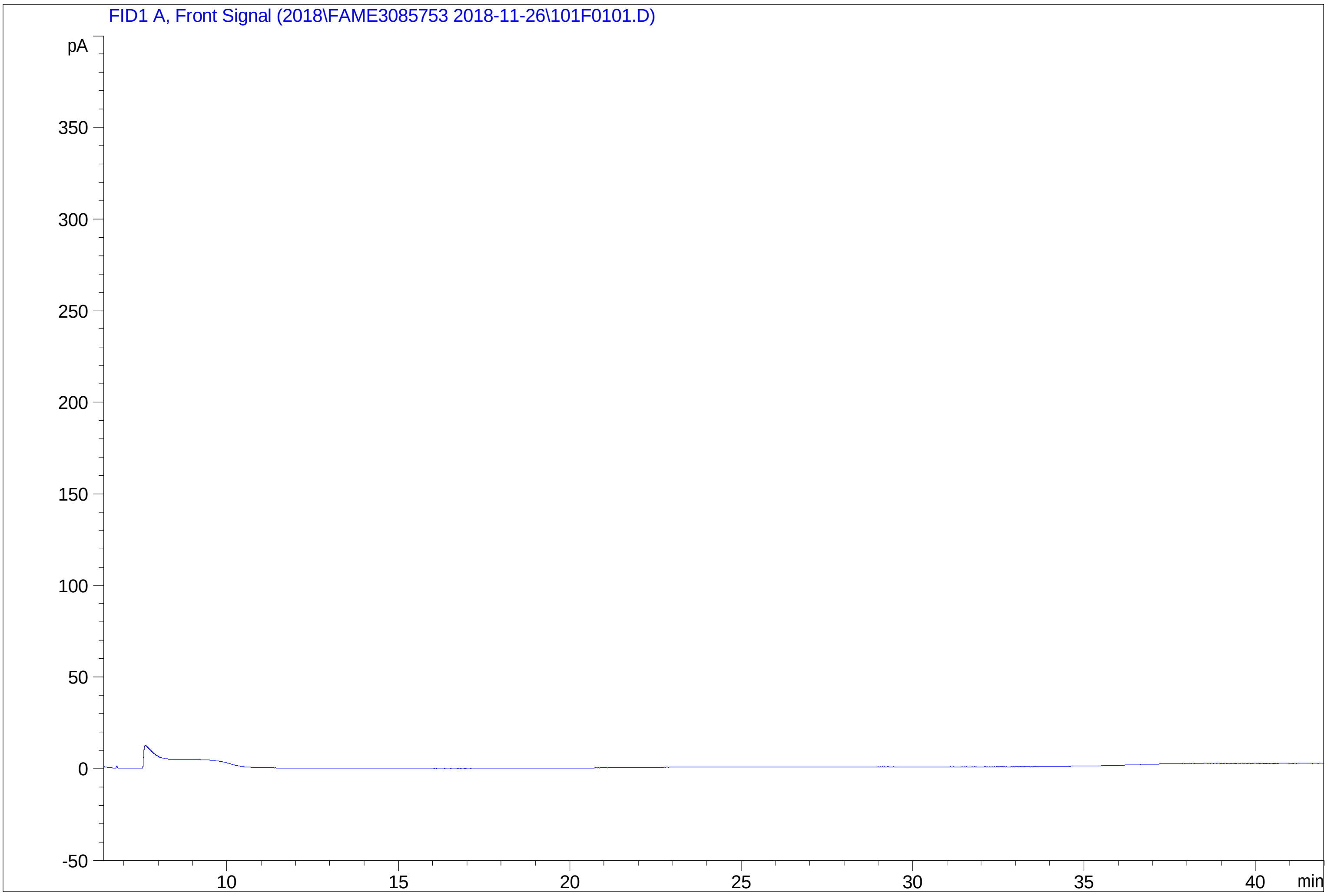

===

Area Percent Report

===

Sorted By : Signal
Calib. Data Modified : 11/26/2018 7:23:18 AM
Multiplier : 1.0000
Dilution : 1.0000
Do not use Multiplier & Dilution Factor with ISTDs

Signal 1: FID1 A, Front Signal

Peak #	RetTime [min]	Type	Width [min]	Area [pA*s]	Area %	Name
1	6.513		0.0000	0.00000	0.00000	C4:0
2	6.908		0.0000	0.00000	0.00000	C6:0
3	7.605		0.0000	0.00000	0.00000	C8:0
4	8.709		0.0000	0.00000	0.00000	C10:0
5	9.430		0.0000	0.00000	0.00000	C11:0
6	10.274		0.0000	0.00000	0.00000	C12:0
7	11.258		0.0000	0.00000	0.00000	C13:0
8	12.412		0.0000	0.00000	0.00000	C14:0
9	13.499		0.0000	0.00000	0.00000	C14:1
10	13.794		0.0000	0.00000	0.00000	C15:0
11	15.129		0.0000	0.00000	0.00000	C15:1
12	15.487		0.0000	0.00000	0.00000	C16:0
13	16.840		0.0000	0.00000	0.00000	C16:1
14	17.523		0.0000	0.00000	0.00000	C17:0
15	18.917		0.0000	0.00000	0.00000	C17:1
16	19.592		0.0000	0.00000	0.00000	C18:0
17	20.400		0.0000	0.00000	0.00000	C18:1n9t
18	20.752		0.0000	0.00000	0.00000	C18:1n9c
19	21.649		0.0000	0.00000	0.00000	C18:2n6t
20	22.453		0.0000	0.00000	0.00000	C18:2n6c
21	23.447		0.0000	0.00000	0.00000	C20:0
22	23.629		0.0000	0.00000	0.00000	C18:3n6
23	24.343		0.0000	0.00000	0.00000	C18:3n3
24	24.499		0.0000	0.00000	0.00000	C20:1
25	25.311		0.0000	0.00000	0.00000	C21:0
26	26.244		0.0000	0.00000	0.00000	C20:2
27	27.344		0.0000	0.00000	0.00000	C22:0
28	27.587		0.0000	0.00000	0.00000	C20:3n6
29	28.450		0.0000	0.00000	0.00000	C20:3n3
30	28.570		0.0000	0.00000	0.00000	C22:1n9
31	28.660		0.0000	0.00000	0.00000	C20:4n6
32	29.582		0.0000	0.00000	0.00000	C23:0
33	30.770		0.0000	0.00000	0.00000	C22:2
34	31.294		0.0000	0.00000	0.00000	C20:5n3
35	32.128		0.0000	0.00000	0.00000	C24:0
36	33.770		0.0000	0.00000	0.00000	C24:1
37	37.878		0.0000	0.00000	0.00000	C22:6n3
Totals :				0.00000	0.0000	

Uncalibrated Peaks: n.a.

2 Warnings or Errors :

Warning : Calibration warnings (see calibration table listing)
Warning : Calibrated compound(s) not found

===

Compound-related custom fields:

*** End of Report ***
